# Unraveling transformation of follicular lymphoma to diffuse large B-cell lymphoma

**DOI:** 10.1371/journal.pone.0212813

**Published:** 2019-02-25

**Authors:** Julia González-Rincón, Miriam Méndez, Sagrario Gómez, Juan F. García, Paloma Martín, Carmen Bellas, Lucía Pedrosa, Socorro M. Rodríguez-Pinilla, Francisca I. Camacho, Cristina Quero, David Pérez-Callejo, Antonio Rueda, Marta Llanos, José Gómez-Codina, Miguel A. Piris, Santiago Montes-Moreno, Carmen Bárcena, Delvys Rodríguez-Abreu, Javier Menárguez, Luis de la Cruz-Merino, Silvia Monsalvo, Consuelo Parejo, Ana Royuela, Ivo Kwee, Luciano Cascione, Alberto Arribas, Francesco Bertoni, Manuela Mollejo, Mariano Provencio, Margarita Sánchez-Beato

**Affiliations:** 1 Lymphoma Research Group, Medical Oncology Department, Instituto de Investigación Sanitaria Puerta de Hierro-Segovia de Arana, Madrid, Spain; 2 Centro de Investigación Biomédica en Red de Cáncer (CIBERONC),Madrid, Spain; 3 Medical Oncology Department, Hospital Universitario Puerta de Hierro, Madrid, Spain; 4 Pathology Department, Hospital MD Anderson Cancer Center, Madrid, Spain; 5 Pathology Department, Hospital Universitario Puerta de Hierro, Madrid, Spain; 6 Pathology Department, Fundación Jiménez Díaz, Madrid, Spain; 7 Pathology Department, Hospital Universitario de Getafe, Madrid, Spain; 8 Medical Oncology Department, Hospital Universitario Virgen de la Victoria, Malaga, Spain; 9 Medical Oncology Department, Hospital Costa del Sol, Malaga, Spain; 10 Medical Oncology Department, Hospital Universitario de Canarias, Santa Cruz de Tenerife, Spain; 11 Medical Oncology Department, Hospital Universitari i Politècnic La Fe, Valencia, Spain; 12 Pathology Department/Translational Hematology Group, Hospital Universitario Marqués de Valdecilla/IDIVAL, Santander, Spain; 13 Pathology Department, Hospital Universitario 12 de Octubre, Madrid, Spain; 14 Medical Oncology Department, Hospital Universitario Insular de Gran Canaria, Las Palmas de Gran Canarias, Spain; 15 Pathology Department, Hospital General Universitario Gregorio Marañón, Madrid, Spain; 16 Medical Oncology Department Hospital Universitario Virgen Macarena, Seville, Spain; 17 Hematology Department, Fundación Jiménez Díaz, Madrid, Spain; 18 TIC Unit- Medical Oncology Department, Instituto de Investigación Sanitaria Puerta de Hierro- Segovia de Arana, Madrid, Spain; 19 Clinical Biostatistics Unit, Instituto de Investigación Sanitaria Puerta de Hierro-Segovia de Arana, Madrid, Spain; 20 Institute of Oncology Research (IOR), Belinzona, Switzerland; 21 Universitá della Svizzera Italiana (USI), Lugano, Switzerland; 22 Dalle Molle Institute for Artificial Intelligence (IDSIA), Belinzona, Switzerland; 23 Swiss Institute of Bioinformatics (SIB), Belinzona, Switzerland; 24 Oncology Institute of Southern Switzerland (IOSI), Belinzona, Switzerland; 25 Pathology Department, Hospital Virgen de la Salud, Toledo, Spain; Institut de recherches cliniques de Montreal, CANADA

## Abstract

Follicular lymphoma (FL) is an indolent but largely incurable disease. Some patients suffer histological transformation to a more aggressive subtype with poorer prognosis. This study aimed to improve our understanding of the genetics underlying FL histological transformation, and to identify genetic drivers or promoters of the transformation by elucidating the differences between FL samples from patients who did and did not transform. We conducted targeted massive parallel sequencing of 22 pre-transformed FL/transformed diffuse large B-cell lymphoma pairs and 20 diagnostic samples from non-transformed FL patients. Additionally, 22 matched samples from 11 transformed FL patients (pre-transformed FL and diffuse large B-cell lymphoma) and 9 non-transformed FLs were studied for copy number variation using SNP arrays. We identified recurrently mutated genes that were enriched at transformation, most notably *LRP1B*, *GNA13* and *POU2AF1*, which have roles in B-cell differentiation, GC architecture and migration. Mutations in *POU2AF1* might be associated with lower levels of expression, were more frequent in transformed FLs, and seemed to be specific to transformed- compared with *de novo-*diffuse large B-cell lymphomas. Pre-transformed FLs carried more mutations per sample and had greater subclonal heterogeneity than non-transformed FLs. Finally, we identified four mutated genes in FL samples that differed between patients who did and did not transform: *NOTCH2*, *DTX1*, *UBE2A* and *HIST1H1E*. The presence of mutations in these genes was associated with shorter time to transformation when mutated in the FL biopsies. This information might be useful for identifying patients at higher risk of transformation.

## Introduction

Follicular lymphoma (FL) is an indolent, but typically incurable disease with a long natural history. FL patients respond to a variety of treatments and have a median overall survival (OS) of >10 years in the immunochemotherapy era [1], but the outcome is variable: some patients do not need treatment, while others follow a more aggressive course characterized by interspersed episodes of remission and relapse, associated with lower sensitivity to therapy [2,3]. The reported frequency of histological transformation (HT) into a more aggressive lymphoma (transformed FL or tFL), most commonly diffuse large B-cell lymphoma (DLBCL), varies markedly, from 30% to 60% of patients. HT has been associated with poor prognosis, and in more than half of cases, occurred within the first year of follow-up [3,4].

Transformation has previously been associated with the activation of the *MYC* oncogene and the inactivation of tumor suppressor genes, such as *CDKN2A/B* and *TP53* [5,6]. More recently, several studies have broadened our knowledge of the genetic alterations associated with HT, revealing, among others, two genes involved in the control of immune recognition by cytotoxic T lymphocytes and NK cells: *B2M* [5,7] and *CD58* [5,7,8]. However, additional studies are needed to clarify the process and to identify biomarkers for predicting transformation.

Recent research on FL suggests that two patterns of tumor evolution drive transformation: the linear and branching/divergent modes [7–10]. HT seems more frequently to follow a divergent pattern of evolution.

In this study, we aimed to improve our knowledge of the genetic aberrations associated with transformation, and to identify genetic alterations associated with transformation by elucidating the differences between FL samples from patients who did and did not transform.

## Materials and methods

### Patients and samples

We collected paired formalin-fixed, paraffin-embedded tissue (FFPET) samples, consisting of paired FL (pre-tFL) and FL transformed to DLBCL (tFL) from 22 transformed patients as well as 20 non-transformed FL (ntFL, median follow-up 11.5 years) diagnostic samples. We obtained the germinal DNA of eight patients from oral mucosa or other non-neoplastic biopsies. The research project was approved by the Ethics Committee of Hospital Universitario Puerta de Hierro-Majadahonda (PI-67/14), and was conducted in accordance with the Declaration of Helsinki. Samples and clinical data were collected (Supplementary Information (SI), S1 Table and S1 Fig), processed and stored according to quality protocols, ensuring the safety and confidentiality of donors’ data. Patients agreed to participate in the study and gave written informed consent. Informed consent for samples previous to 2007 was waived by the Ethics Committee. Transfer of material and data from other collaborator biobanks was approved by the corresponding ethical and external scientific committees according to Spanish legislation (*Ley 14/2007 de Investigacion Biomedica* and *Real Decreto 1716/2011*) and anonymously transferred to our center. All samples were reviewed by expert hematopathologists (MMo and CB) upon arrival to confirm their diagnosis and to select enriched tumoral areas, as necessary.

An additional cohort of 48 samples from patients with *de novo* DLBCL including GCB and ABC type cases (data not shown) was included to enable comparison.

### Target resequencing

The genes included in the panels (S2 Table) are involved in lymphomagenesis pathways. They were selected based on the results of previous studies [7,8,11].

Genomic DNA was extracted with a truXTRAC FFPE DNA Kit (Covaris, Woburn, MA, USA) or a QIAamp DNA FFPE Tissue Kit (Qiagen, Manchester, UK) in accordance with the manufacturers’ instructions.

The HaloPlex Target Enrichment custom panel was designed using the SureDesign web-based tool (earray.chem.agilent.com/suredesign/) (Agilent Technologies, Santa Clara, CA, USA). The design covered all coding exons and 10 flanking bases at the 3´ and 5´ ends of the 167 selected genes (S2 Table). The target regions (according to Human Assembly GRCh37/hg19) were captured using a HaloPlex Target Enrichment kit (Agilent), following the manufacturer’s instructions. Samples enriched using this custom panel are indicated in S3 Table. Sequencing was performed using the Ion Proton system (Thermo Fisher Scientific, Waltham, MA, USA) at NIMGenetics Genómica y Medicina, S.L. (Madrid, Spain) to achieve a minimum read depth of 500x (S3 Table). Nine tumoral and two non-tumoral samples (from seven patients) were analyzed in this way (S3 Table and S2 Fig), but the system was eventually discarded because the large quantity of DNA needed and the large amount of non-targeted sequencing data generated meant that a substantial percentage of samples could not be used.

A SureSelect target enrichment custom panel was then designed using the SureDesign (Agilent) web-based tool. The design covered all coding exons of 186 genes. Captured libraries were diluted to 11 pM for Illumina clustering, and paired-end sequencing was performed on MiSeq (Illumina Inc., San Diego, CA, USA). 110 genes were common to the two designs and are referred to as the common gene set (CGS).

Two independent analyses were performed. The first was done with the tools available in the Variant Reporter instrument (Illumina) or with the Ion Reporter Software (Thermo Fisher). Sequencing data were also aligned using a BWA aligner, the duplicates being marked using a Picard tool (Broad Institute, Cambridge, MA, USA). Single-nucleotide variants were predicted using VarScan [12] then annotated using Annovar [13].

The TruSeq Custom Amplicon v1.5 Low Input Library for dual-strand sequencing (Illumina) was used to validate mutations in selected samples. The probes for this custom panel were designed with DesignStudio (Illumina) and consisted of 1399 amplicons with an average size of 175 bp and a cumulative target region of 140 kb. The target genes are listed in S2 Table.

Target enrichment was performed in FFPET-extracted DNA in accordance with the manufacturer’s instructions (TruSeq Amplicon—Cancer Panel Library Preparation Guide; March 2016; Illumina). The pooled libraries were sequenced with a MiSeq Reagent Kit V2 (paired-end, 2x151) in a MiSeq instrument (Illumina), as described in the manufacturer’s protocol.

The variant lists obtained were analyzed by *Excel* filtering and visualization using the Integrative Genome viewer (IGV) tool [14]. The Genome Analysis Toolkit annotated the SNPs available at dbSNP 138 (hg19), and those reported by the 1000 Genomes Project were filtered out. Missense, nonsense, frameshift and splicing mutations were selected.

Data are available in the SRA (accession number SRP121648).

### SNP arrays

The DNAs were first subjected to the Infinium HD FFPE DNA Restore Protocol (Illumina) and up to 200 ng DNA per sample were hybridized for the SNP analysis. Genotyping was conducted using the Illumina HumanOmni2.5 BeadChip according to the manufacturer's protocols (Illumina). The BeadChip images were scanned on the iScan system and the data (genotypes, log R ratio (LRR) and B allele frequency (BAF)) were extracted into Illumina's Genome Studio v2011.1. The software’s default settings, developed by Illumina for genotype calling, were applied with the cluster file.

Raw LRR values were normalized probe-wise using the median of each batch. Segmentation was conducted using Fast First Derivative Segmentation after normalizing each profile [15]. Copy number segments overlapping known copy number variants [16] were excluded from further analysis. Segments with LRR values <-0.2 were considered to be ‘lost’; segments with LRR values >0.2 were considered to be ‘gained’. Focal recurrent aberrations were detected using GISTIC 2.0 [17]. Recurrent minimal common regions (MCRs) were defined using the algorithm described by Lenz et al. [18].

### Sanger sequencing

Sanger sequencing was used to verify the published *POU2AF1* mutations in cell lines. Primers (FW-CCAATTCCAGACCAAGCTGT and RV-AGGAAACAGCCTCAGCTCAA) were designed using Primer 3 (http://frodo.wi.mit.edu/prim). PCR products were cleaned using ExoSAP-IT (Affymetrix, Santa Clara, CA, USA) and sequenced using ABI Prism BigDye terminator v3.1 (Life Technologies). Sequencing reactions were run on an ABI-3100 Avant Genetic Analyzer (Life Technologies).

### Western blot

Protein was extracted from Raji, RL, SU-DHL6 and OCI-LY19 using radioimmunoprecipitation assay (RIPA) lysis buffer supplemented with protease and phosphatase inhibitors. Western blot analysis was conducted following standard protocols.

The primary antibodies used were BOB1 (SP92) from Ventana Medical Systems, Inc. (Oro Valley, AZ, USA) and α-tubulin (T-6074) from Sigma-Aldrich (San Luis, MO, USA). The secondary antibodies used were labeled with Alexa 680 nm or Alexa 800 nm. Western blots were quantified using the Image Studio program (LI-COR, Lincoln, NE, USA) and graphs generated using GraphPad Prism7 (GraphPad Software, La Jolla, CA, USA).

### Quantitative RT-PCR

Total RNA was extracted and purified using an RNeasy Mini-Kit (Qiagen, Hilden, GE, USA), and quantified with a Nanodrop instrument (Thermo Fisher Scientific). cDNA was synthesized from 500-ng DNaseI-treated RNA (RNase-Free DNase Set; Qiagen) with Affinity Script Multi-Temp Reverse Transcriptase (Agilent) and random primers.

The level of expression of POU2AF1 transcript was determined by real-time quantitative PCR using splice junction-specific primers (the forward primer was designed in exon 2 and the reverse one in exon 1) combined with LightCycler 480 SYBR green (Roche, Basel, Switzerland). Primers were designed with the Primer 3 tool: forward (5’-TTCCTTCACTGGCTGCTTCA-3’) and reverse (5’-AGGAAACAGCCTCAGCTCAA-3’).

Values were normalized with respect to the expression of succinate dehydrogenase complex flavoprotein subunit A (SDHA) as an endogenous control. Measurements were taken from three cultures of each cell line. Each of them was performed in triplicate. PCR reactions were performed and analyzed using the LightCycler 480 (Roche), software release 1.5.0, Advance Relative Quantification method. PCR products were checked by melt curve genotyping, and electrophoresis on 2% agarose gels.

### Statistical analysis

Associations between factors were assessed using Fisher’s exact test. Mann-Whitney U-test was used to analyze the differences in *POU2AF1* gene expression between mutated and non-mutated cell lines.

Overall survival (OS) was calculated from the date of diagnosis to the date of death or last follow-up, whichever occurred first. Time to transformation (TTT) was calculated from the date of FL diagnosis to the date of HT. To evaluate TTT, the survival functions were estimated by the Kaplan–Meier method. The log-rank test was used to compare patient groups and univariate and multivariate Cox regression models were used to assess associations with TTT. Hazard ratios with 95% confidence intervals were estimated for each parameter. The results were shown graphically and by transformation-free survival at several follow-up times. We then computed the points for each predictive variable (mutated gene) as the rounded value of the quotient resulting from dividing the coefficient of each variable by the lowest coefficient. The risk score was calculated for each patient based on the sum of individual points of each predictive variable included in the final model.

For all analyses, values of p < 0.05 were considered significant. Statistical analyses were performed using STATA v14.2 (StataCorp LLC, College Station, TX, USA), IBM SPSS Statistics v19 (IBM Corp., Armonk, NY, USA) or GraphPad Prism 7 (GraphPad Software, San Diego, CA, USA)

## Results

### Recurrent genetic alterations in transformed follicular lymphoma

**Fig 1:**
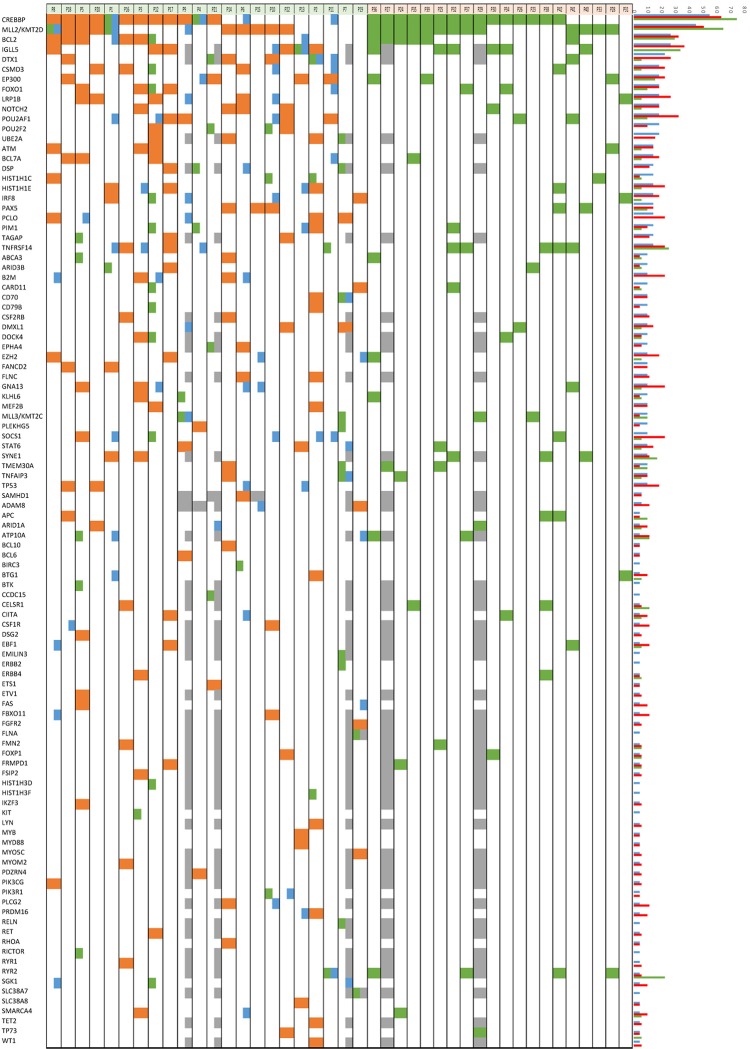
Distribution of genes recurrently mutated in 22 pre-tFL/tFL and 20 ntFL cases. Columns represent individual patients, and rows denote specific genes. The bar graph shows the frequency of mutations found in each gene. Green: FL-specific; orange: FL & tFL (shared), blue: tFL-specific; grey: not done.

We conducted targeted massive sequencing analysis in paired samples of pre-tFL and transformed DLBCL (tFL) from 22 patients (S1 and S2 Figs, S1 Table). Median coverage was 543x, with a median of 96% of targeted bases covered by >50x. These values allowed us to identify minor mutated clones with confidence down to a variant allele frequency (VAF) of 5% (some of them validated with the TruSeq panel, S4 Table). In pre-tFL samples, the most recurrently mutated genes (considering only missense, non-sense, frameshift and splicing mutations) were *CREBBP* (55%), *KMT2D* (45%), *IGLL5* (27%), *BCL2* (27%) and *DTX1* (23%) (Fig 1, S4 and S5 Tables). In the CGS (S2 Table), there was a median frequency of six non-synonymous mutations (range, 2–14).

In tFL samples, the most recurrently mutated genes were *CREBBP* (64% of cases), *BCL2* (32%), *KMT2D* (50%), *IGLL5* (37%), *POU2AF1* (32%), *DTX1* (26%), *LRP1B* (27%), and *B2M*, *CSMD3*, *EP300*, *GNA13*, *HISTH1E*, *PCLO*, *SOCS1* and *TNFSRF14* (23% each) (Fig 1, S4 and S5 Tables). In this case, there was a median of eight mutations (range, 4–16).

Some genes were more frequently mutated in tFL than in matched pre-tFL samples, although the differences were not statistically significant. The most relevant were *SOCS1* (pre-tFL = 9.1% *vs*. tFL = 22.7%), *GNA13* (9.1% *vs*. 22.7%), *B2M* (9.1% *vs*. 22.7%), *LRP1B* (18.2% *vs*. 27.2%) and *POU2AF1* (18.2% *vs*. 31.8%).

Multiple mutations were found in several genes known to be targets of aberrant somatic hypermutation (aSHM) (e.g., *BCL2*, *PIM1*, *SOCS1*, *IGLL5* and *DTX1*) in pre-tFL and tFL samples. aSHM occurs in >50% DLBCLs that arise *de novo* [19,20], but is rare in FL [7,21]. This pattern was clearer in the transformed samples, but was also present earlier in some pre-tFL samples (S4 and S7 Tables). The number of mutations in these genes increased slightly at transformation (S7 Table).

We also studied copy number variations (CNVs) using SNP arrays in 22 matched samples (pre-tFL and tFL) from 11 transformed patients. As the DNA analyzed came from FFPETs, we could not obtain high-resolution results, but our findings were interesting nevertheless (Table 1, S3 Fig). Loss of chromosomal region 6q16.1-6q23.3 was a recurrent event in transformed FL patients, being found in 3/11 patients following the diagnosis of pre-tFL. Two patients conserved the loss from the pre-transformed to the transformed sample, whereas in the other we did not detect this loss at transformation. Another patient lost this region at transformation. Genes of interest, such as *TNFAIP3*, *MYB*, *SGK1* and *PRDM1*, were located in the minimal common deleted region and has been previously described as being associated with FL transformation [22]. The 1p36.23-p36.32 region, which includes *PLEKHG5*, *PRDM16*, *TNFSRF14* and *TP73*, was lost in 1/11 pre-tFL and 2/11 tFL samples, all of which were from different patients. Losses of part of this region have also been described in *de novo* DLBCL [23]. Finally, 17p13.1 (where *TP53* is located) was deleted after transformation in three cases. The deletion was also detected in the pre-tFL biopsy of one patient [22]. Another notable finding was the loss of 2q22.1 in 3/11 tFL samples. This region contains the *LRP1B* gene, which was mutated in 6/22 (27%) tFL samples, and differs from those with the deletion. This finding was further evidence of the potential importance of this gene in transformation.

**Table 1:** Recurrent CNV alterations in 11 pre-tFL / tFL and 9 ntFL cases.

Region	pre-tFL	tFL	ntFL	CNV	Genes included in MCR
**1p36.23-1p36.32**	9.1%	18.2%	22.2%	LOSS	*PRAK7*, *TNFRSF25*, *TNRSF9*, *PLEKHG5*, *PRDM16*, *TNFRSF14*, *TP73*
**6q16.1-6q23.3**	27.3%	27.3%	0.0%	LOSS	*EPHA7*, *PRDM1*, *SGK1*, *TNFAIP3*, *MYB*
**8p1.23-8p21.3**	0.0%	36.4%	11.1%	LOSS	*FGFR1*, *UNC5D*, *TNFRSF10B*, *ATP6V1B2*, *TNFRSF10A*
**15q15.4-15q21.1**	0.0%	18.2%	0.0%	LOSS	*PLA2G4B*, *TGM7*, *B2M*
**17p13.1**	9.1%	18.2%	0.0%	LOSS	*TP53*
**2q22.1**	0.0%	27.3%	0.0%	LOSS	*LRP1B*

CNV: copy number variation; FL: follicular lymphoma; tFL: transformed FL; ntFL: non-transformed FL; MCR: minor common region.

**Fig 2:**
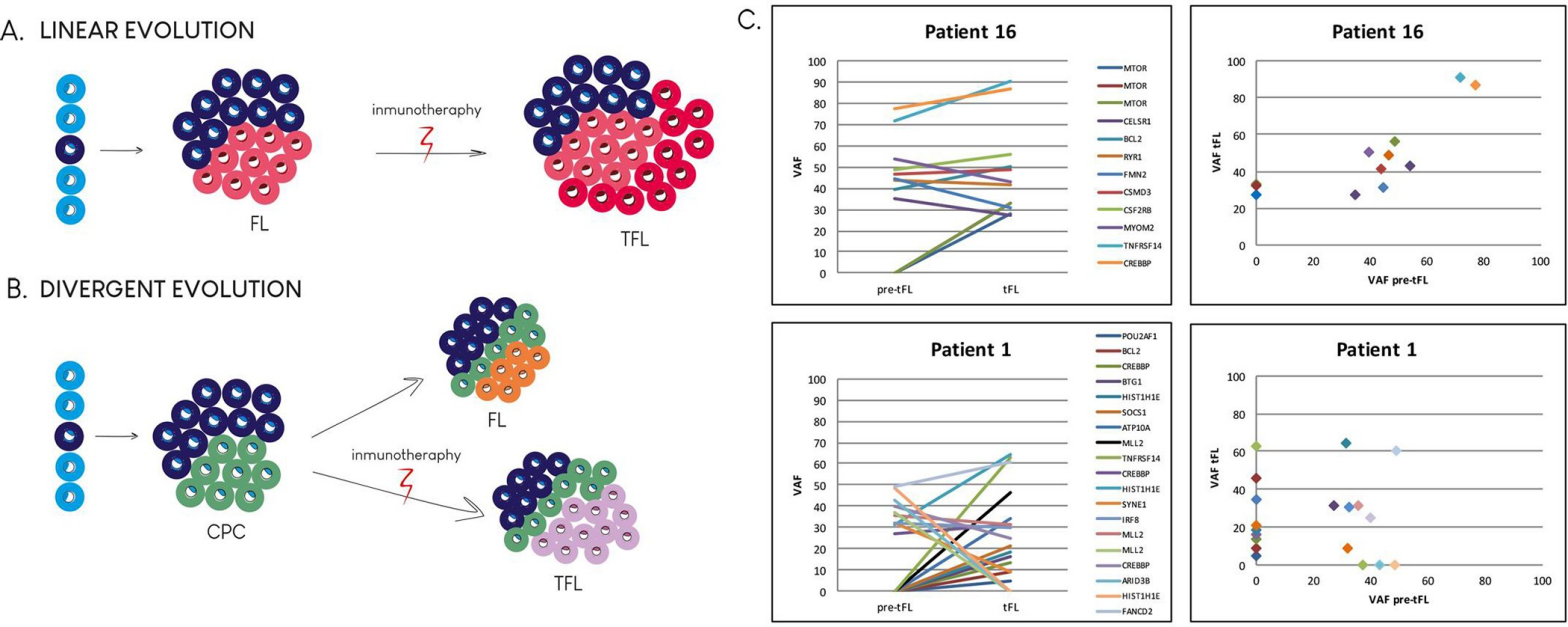
Patterns of evolution of follicular lymphoma to transformed follicular lymphoma for two model cases. Transformed follicular lymphoma (tFL) can arise through two evolutionary modes: linear (**A**) or divergent (**B**). In the linear model (A & C, patient 16) the tFL-dominant clone originates from the FL (pre-tFL) after acquiring additional mutations. In the divergent model (B & C, patient 1), the pre-tFL and tFL are derived from a common precursor and acquire independent and distinct mutations. For more examples, see S4 Fig. VAF: variant allele frequency. CPC: common precursor cell.

We also analyzed the subclonal composition and patterns of evolution during transformation. Our design took advantage of known recurrently mutated genes (186 genes, analyzed in most of the patients), and the greater depth achievable (median depth >500x) than with WES (20-60x) [7,8,10], which allowed us to detect minor, but informative mutated clones, and thereby determine the evolutionary patterns. All sample pairs shared common mutations, but in several cases, pre-tFL and tFL biopsies both had a fraction of specific mutations. We concluded linear evolution for patients who conserved all the mutations identified in the pre-tFL biopsy that acquired new mutations in the tFL sample and divergent evolution for those who had independent mutations, even though they shared a pool of mutations (Fig 2 and S4 Fig).

### Recurrent splicing mutations in *POU2AF1*

The *POU2AF1* gene (also known as BOB1/OBF.1) was recurrently mutated in tFL samples (31.8%) at a higher frequency than in pre-tFL biopsies (18.2%) (Fig 1, S4 and S5 Tables). The mutations were located in the 3-bp exon 1 splice donor site. They were validated as somatic in two patients from whom we had obtained germinal DNA (P9 and P22). Additionally, two of the *POU2AF1*-mutated tFL cases had mutations in the *POU2F2* gene (known as OCT2). When we analyzed the *POU2AF1* gene in a group of 48 *de novo* DLBCL samples, including germinal center B-cell like (GCB) and non-GCB cases classified according to cell of origin, we found only one mutated case in the series (personal unpublished observations), suggesting that this alteration might be specific to DLBCL of transformed patients (Fisher’s exact test p = 0.001).

We analyzed the putative effect of these mutations *in silico* using the Splicing Prediction Module available within the Alamut Visual (Interactive Biosoftware, Rouen, France), reporting that skipping exon 1 is very likely for mutations in positions chr11: 111249885 (A/T) and chr11: 111249886 (C/T) (S6 Table).

We then evaluated the effect of these splicing-site mutations on gene expression levels. To this end, we identified two B-cell lymphoma-derived cell lines, SU-DHL6, with a mutation in chr11: 111249884 T/A (found in two patients, S4 Table), and OCI-LY19, with a mutation in chr11: 111249886 C/T (found also in two patients), and two wild type cell lines, Raji and RL. We performed western blot and RT-PCR to analyze *POU2AF1* gene expression. For RT-PCR we used primers designed for each of the alternative POU2AF1 transcripts but only the POU2AF1-001 transcript (the canonical one) was detected by RT-PCR. We then performed quantitative RT-PCR (qPCR) using primers designed in exon 1 and exon 2 to evaluate the effect of the exon 1 splice donor site mutations on POU2AF1-mRNA expression. The results showed that the levels of protein (Mann-Whitney U test, P = ns) and mRNA (Mann-Whitney U test, P = 0.009) in the two *POU2AF1*-mutated cell lines were lower than in the wild-type cell lines (S5 Fig).

### Genetic lesions associated with transformation

When we analyzed the entire cohort of 42 FL samples (22 pre-tFL and 20 ntFL) we found that the most recurrently mutated genes were *CREBBP* (67% of cases) and *KMT2D* (55%), followed by *IGLL5* (35%), *BCL2* (29%), *TNFRSF14* (17%), *EP300* (17%), *DTX1* (17%), *FOXO1* (17%) and *POU2AF1* (14%) (Fig 1, S4, S5 and S7 Tables). These results were similar to those of other series [8].

The analysis of the impact of mutated genes on FL patients’ clinical outcome showed that *IRF8*, *BCL7A* and *TP53* mutations were associated with shorter OS (Table 2).

**Table 2:** Multivariate Cox regression analysis of overall survival by mutational status.

	HR	p	95% CI	
***BCL7A***	2.074	0.051	0.996	4.320
***IRF8***	5.212	0.017	1.345	20.191
***TP53***	5.818	0.033	1.148	29.502

Multivariate analyses considered the mutated genes found to be significant in univariate analyses. HR: hazard ratio; CI: confidence interval.

We next compared the number of mutated genes per sample in the pre-tFL with ntFL, and found significantly more mutated genes in the pre-tFL than in the ntFL samples (means of 6.4 in ntFL *vs*. 9.0 in pre-tFL; p = 0.031). Additionally, the heterogeneity in the patterns of mutations was greater in pre-tFL biopsies: 48 genes were recurrently mutated in the pre-tFL samples, compared with 23 genes in the ntFL samples (S4 and S5 Tables and S6 Fig). We also found that the mean VAF of all mutations in pre-tFL samples was significantly lower than in ntFL (VAF = 27.77% ± 0.895 in pre-tFL *vs*. 34.66% ± 1.178 in ntFL; p < 0.001). In fact, 35% of the mutations in pre-tFL samples have VAF < 20% compared with 20% of the mutations in ntFL samples (S6 Fig), suggesting that a notable proportion of somatic mutations in pre-tFL samples are not present in the dominant tumoral clone.

The SNP array analysis (Table 1 and S3 Fig) revealed a loss of the chromosomal region 6q16.1-6q23.3 (containing *SGK1* and *TNFAIP3* genes), which seems to be specific to or, at least, more frequent in transformed patients (ntFL: 0/9; pre-tFL: 3/11). The 1p36.23-p36.32 region was lost in 2/9 ntFL and 1/11 pre-tFL samples, as previously described, and therefore, it does not seem to be associated with the transformation process.

M7-FLIPI integrates the mutation status of seven genes (*EZH2*, *ARID1A*, *MEF2B*, *EP300*, *FOXO1*, *CREBBP* and *CARD11*), the FL International Prognostic Index (FLIPI) and ECOG performance status, and it was developed to identify patients at high risk of poor outcome to first-line therapy [24]. Although it was not established for the purpose of predicting patients with a higher risk of HT, we still calculated the m7-FLIPI score (low *vs*. high risk) for the patients included in this series (S1 Table) and performed Cox regression analysis to test its impact on transformation. We found a significant association of this score with TTT (HR = 2.692, p = 0.032).

**Fig 3:**
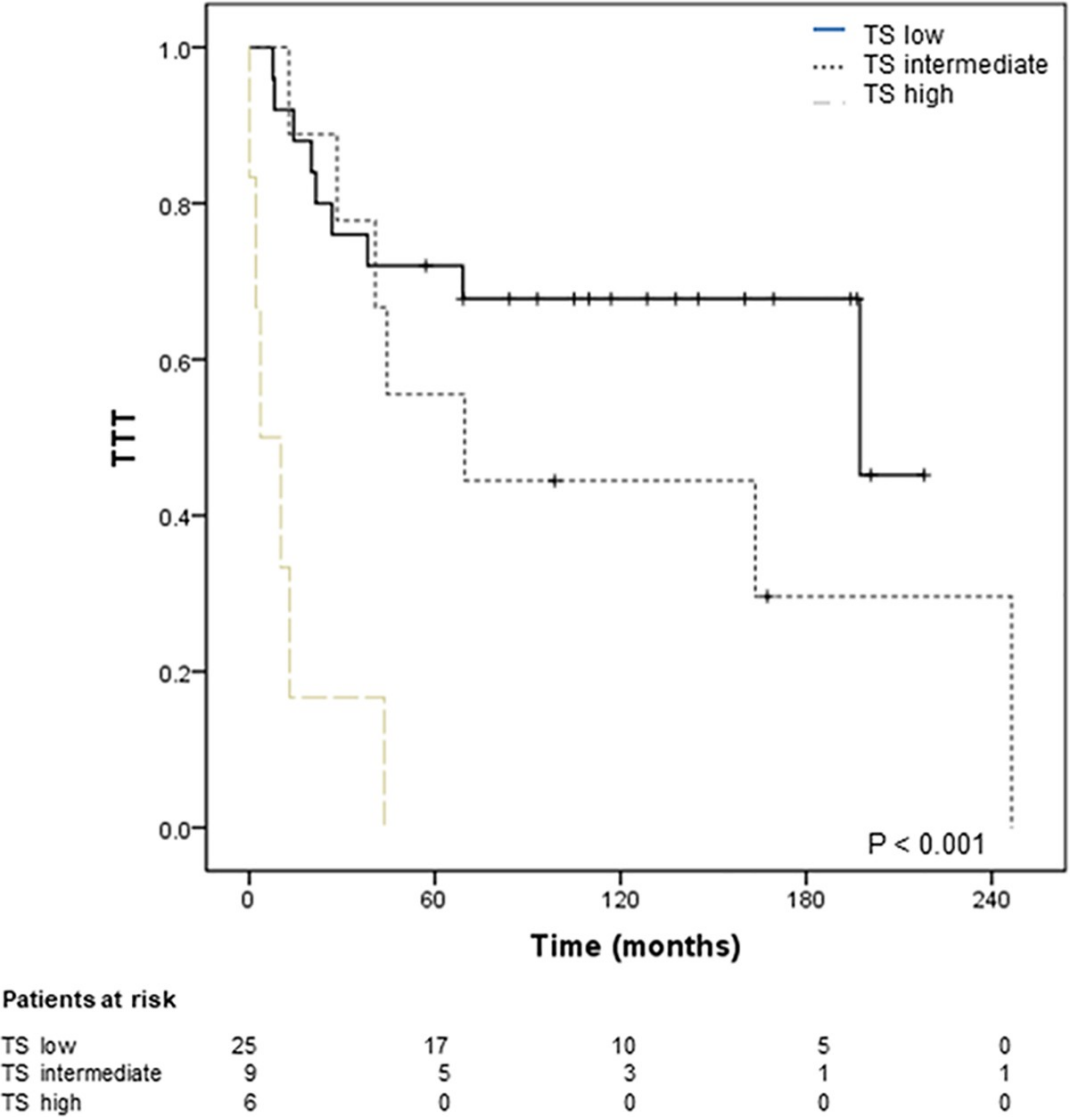
Estimates of time to transformation of patients classified by transformation risk score. Time to transformation (TTT) according to the score groups calculated for each patient based on the sum of individual points of each mutated gene significantly associated with transformation in the Cox regression analysis (Table 3) and included in the final model. Forty patients were included in the analysis: 22 suffered histological transformation and 18 did not transform. After 5 years (60 months) 100% of high-risk, 55% of intermediate-risk, and 32% of low-risk patients had transformed. The log-rank p value is shown. TS: transformation risk score.

Finally, we examined our series for genes that were recurrently mutated in pre-tFL and not in ntFL, or more frequently in pre-tFL than in ntFL samples and performed univariate and multivariate Cox regression analyses of the association of their mutational status with TTT. There were four such genes: *UBE2A*, *DTX*, *NOTCH2* and *HISTIH1E* (Table 3). The Kaplan–Meier analyses further support these results. We assigned a risk score according to the regression coefficients of the multivariate analysis for the four genes and categorizing the scores into low (= 0), high (≥ 2) or intermediate groups (Fig 3). After five years, 100% of patients with a high-risk, 55% with an intermediate risk and 32% with a low risk score were transformed.

**Table 3:** Multivariate Cox regression analysis of time to treatment by mutational status.

	HR	p	95% CI		Regression coefficient	Adjusted score
***DTX1***	3.005	0.026	1.139	7.929	1.100	1.0
***HIST1H1E***	4.889	0.010	1.464	16.322	1.587	1.5
***UBE2A***	5.081	0.018	1.329	19.426	1.626	1.5
***NOTCH2***	7.533	0.002	2.069	27.431	2.019	2.0

Multivariate analyses considered the mutated genes found to be significant in univariate analyses. HR: hazard ratio; CI: confidence interval.

## Discussion

In this study, we analyzed paired samples from transformed FL and samples from non-transformed patients by targeted massive parallel sequencing and SNP arrays.

We found some mutated genes became enriched upon transformation. Some of these have already been reported (*SOCS1*, *GNA13*, *B2M*) [7,10], but others (*POU2AF1*, *LRP1B*) have not been highlighted before and could be involved in the transformation (See S8 Table).

Previous studies of GNA13-deficient mice showed increased frequencies of somatic hypermutation at the immunoglobulin VH locus, a phenomenon we found in our series in several genes known to be targets of aSHM and previously described by others [25]. Observations in GNA13-deficient mice have also revealed that changes in this gene could contribute to alterations in germinal center (GC) architecture by affecting B-cell migration behavior [25,26].

Loss of *LRP1B*, by somatic mutation or gene deletion, could also be involved in these processes. This gene is one of those most frequently deleted in human tumors [27] and its inactivation provokes changes in the tumor environment, conferring increased growth and invasive capacity on cancer cells [28].

BOB1 (POU2AF1) is a transcriptional coactivator that interacts with OCT1 (POU2F1) and OCT2 (POU2F2) to promote immunoglobulin transcription, and is essential for B-cell fate determination and GC formation in mice [29]. We identified a hotspot of three nucleotides in the exon 1 splicing donor site that were recurrently mutated in *POU2AF1*, as well as mutations in *POU2F2*. *POU2AF1* mutations have been described before [30], but we made two novel findings about them. First, their frequency is higher in FL samples from transformed patients than in *de novo* DLBCL cases [31,32], suggesting that this alteration might be specific to DLBCLs of transformed patients. Second, *POU2AF1* splicing mutations might reduce its expression levels, thereby compromising its function in B-cell differentiation and/or GC formation. However, this possibility needs to be explored further. One of the *POU2F2* mutations we found was the previously described T223A [33], which alters the DNA-binding capacity of OCT2 in some of its non-canonical target promoters (*HIF1a* and *FCRL3*), leading to their transactivation. BOB1 and OCT2 cooperate to promote transcription of BCL6 and BTK, among other proteins, which are essential for B-cell differentiation and/or GC formation, as demonstrated in mice [34–36]. The recurrence of mutations in *POU2AF1* and *POU2F2*, and the results of other studies [33,36,37], suggest that targeting the OCT2/BOB1 complex might be a therapeutic strategy for the treatment of tFL.

We found two distinct patterns of clonal evolution, as has been described in previous studies [7,8,10]: divergent and linear patterns of evolution in 55% and 45% of patients, respectively. However, we are aware of the limitations of this analysis, which used targeted sequencing rather than whole-exome sequencing. Although the design allowed us to detect minor, but informative mutated clones, this could lead to a bias towards inferring a linear pattern.

Comparison of pre-tFL and ntFL samples revealed greater heterogeneity and a more complex mutational landscape in transformed than in non-transformed patients, as has been previously associated with worse clinical course in CLL and other neoplasias. A noteworthy finding of this study was the association of the presence of mutations in four genes (*NOTCH2*, *DTX1*, *UBE2A* and *HISTIH1E*) with transformation. These genes were more frequently mutated in pre-tFL than in ntFL samples and significantly associated with TTT (Table 3 and Fig 3), although the validity of this association and the possible use of these genes as biomarkers predicting transformation need to be validated in independent prospective series.

DTX1 is an E3 ubiquitin ligase that regulates, among others, Notch proteins. All of the mutations (12 non-synonymous mutations in seven patients), except two splicing mutations, were located in exon 1 (S4 Table), affecting the WWE1 domain of the protein, and have been previously demonstrated to weaken DTX1 function as a negative regulator of Notch [38]. *DTX1* mutations are associated with shorter time to progression and OS in DLBLC [39]. NOTCH2, regulated by DTX1 protein, has been found recurrently mutated in several types of lymphoma, including splenic marginal zone lymphoma [40,41], DLBCL [31,32,42] and FL [30,43]. In FL, mutations in *NOTCH1* and *NOTCH2* are significantly associated with FL HT or the presence of DLBCL zones in the FL tumor [43]. NOTCH2 function has recently been reported to be negatively regulated by BCL6 [44], and BCL6 inhibitors seem to induce NOTCH2, suppressing the growth of FL xenografts *in vivo*. All these findings suggest that NOTCH pathway malfunction could be relevant in FL transformation.

The *UBE2A* gene was mutated in the pre-tFL and tFL samples of four patients. The UBE2A protein is also involved in the ubiquitination process. It is a E2 ubiquitin-conjugating enzyme and is mutated in 10% of DLBCLs of Chinese patients [38]. Finally, *HIST1H1E* is a chromatin-remodeling gene that has been associated with FL transformation [7,10]. We found this gene mutated in five transformed FL patients, in the pre-tFL sample in three of them (one with two mutations), and three additional mutations in two patients in the tFL sample.

In summary, we identified recurrently mutated genes that may be involved in transformation, the most relevant of which (*POU2AF1*, *GNA13* and *LRP1B*) have roles in B-cell differentiation, GC architecture and migration. We observed a more complex mutational landscape in pre-tFL than in ntFL patients. We also identified four genes (*NOTCH2*, *DTX1*, *UBE2A* and *HIST1E1*) whose mutations in FL samples are associated with transformation, and so could be used to predict transformation at the time of FL diagnosis and for following up patients and selecting appropriate clinical management, such as treating patients at higher risk of transformation, rather than watching and waiting. Our findings also highlighted the putative relevance of the Notch pathway in FL HT.

## Supporting information

S1 Fig Temporal evolution and biopsy information for the 22 transformed follicular lymphoma cases. Circle: FL biopsy; Square: transformed FL biopsy; Arrow: last follow up; vertical bar: Exitus.
(TIF)

S2 Fig Diagram of patient samples and sequencing approaches. (TIF)

S3 Fig Graph of copy number variation. ntFL: non-transformed follicular lymphoma; pre-tFL: follicular lymphoma samples from transformed patients; tFL: diffuse large B-cell lymphoma samples from transformed patients.
(TIF)

S4 Fig Clonal evolution of mutations from follicular lymphoma (FL) to diffuse large B-cell lymphoma (transformed FL (tFL)). ntFL: non-transformed FL; pre-tFL: FL samples from transformed patients.
(PDF)

S5 Fig Analysis of BOB1 expression in POU2AF1-mutated and wild-type B-cell lymphoma-derived cell lines. **A)** DNA sequences corresponding to the *POU2AF1* gene in mutated cell lines (SU-DHL6, mutated in chr11: 111249884 T/A; and OCI-LY19 mutated in chr11: 111249886 C/T) and wild-type cell lines (Raji and RL). **B)** Quantitative RT-PCR analysis of POU2AF1-mRNA expression in the cell lines. Graphs represent the means of POU2AF1-mRNA levels and SDs, normalized with respect to SDHA, of three independent mRNA extractions; Mann Whitney two-tailed test, p = 0.009. **C)** Western blot analysis of BOB1 expression in the cell lines. **D)** Graphs show the means and SDs of quantified BOB1 protein levels normalized with respect to α-tubulin expression of four independent protein extractions; Mann Whitney two-tailed test, p = 0.065. ns: not significant; **: p<0.01. Grey circle: expression values corresponding to SU-DHL6 cell line; Black circle: expression values corresponding to OCI-LY19 cell line; Grey square: expression values corresponding to Raji cell line; Black square: expression values corresponding to RL cell line.
(TIF)

S6 Fig Variant allele frequency (VAF) of gene mutations identified in pre-tFL (follicular lymphoma from transformed patients) and ntFL (non-transformed follicular lymphoma) samples. Blue boxes indicate VAFs < 20%.
(TIF)

S1 Table Summary of clinical features of FL patients. (XLSX)

S2 Table Lists of genes included in the different custom panels. (XLSX)

S3 Table Quality and coverage information for patients sequenced in different platforms. (XLSX)

S4 Table SNV and Indel from targeted sequencing with a allelic frequency >5%. (XLSX)

S5 Table Summary of recurrent mutations in the dufferent groups of samples. (XLSX)

S6 Table Alamut predictions for POU2AF1 splicing mutations. (XLSX)

S7 Table Covered region mutation rate. (XLSX)

S8 Table Comparative results summarizing the frequency of gene mutations in paired pre-tFL and tFL samples from FL patients with histologic transformation. (XLSX)
