# Supplementary figures and images for: Unraveling transformation of follicular lymphoma to diffuse large B-cell lymphoma
Source: PLoS One. 2019 Feb 25;14(2):e0212813. doi: 10.1371/journal.pone.0212813 (PMC6388933; doi:10.1371/journal.pone.0212813)

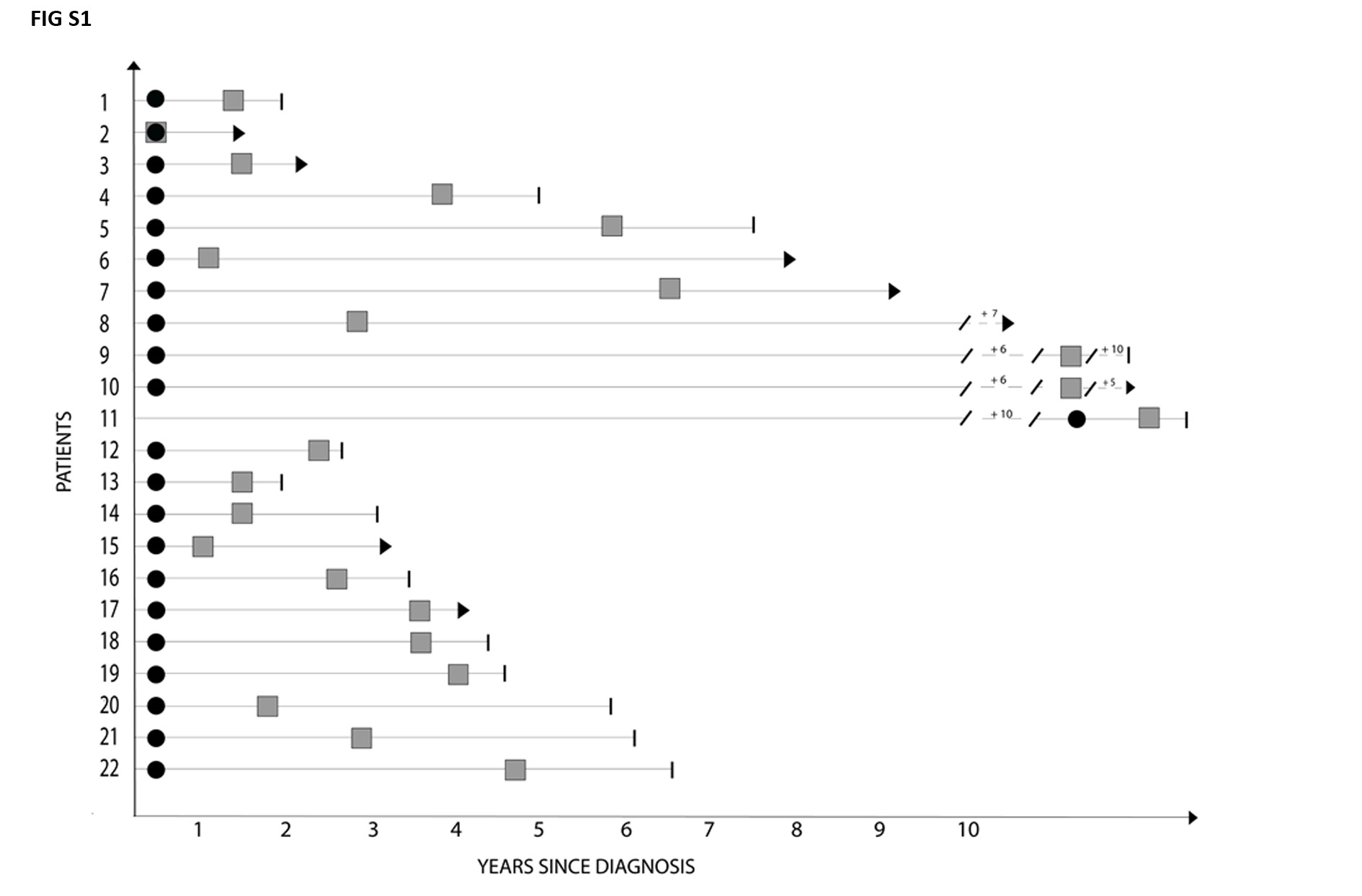

Supplement: S1 Fig — Circle: FL biopsy; Square: transformed FL biopsy; Arrow: last follow up; vertical bar: Exitus. (TIF) [file pone.0212813.s001.tif]

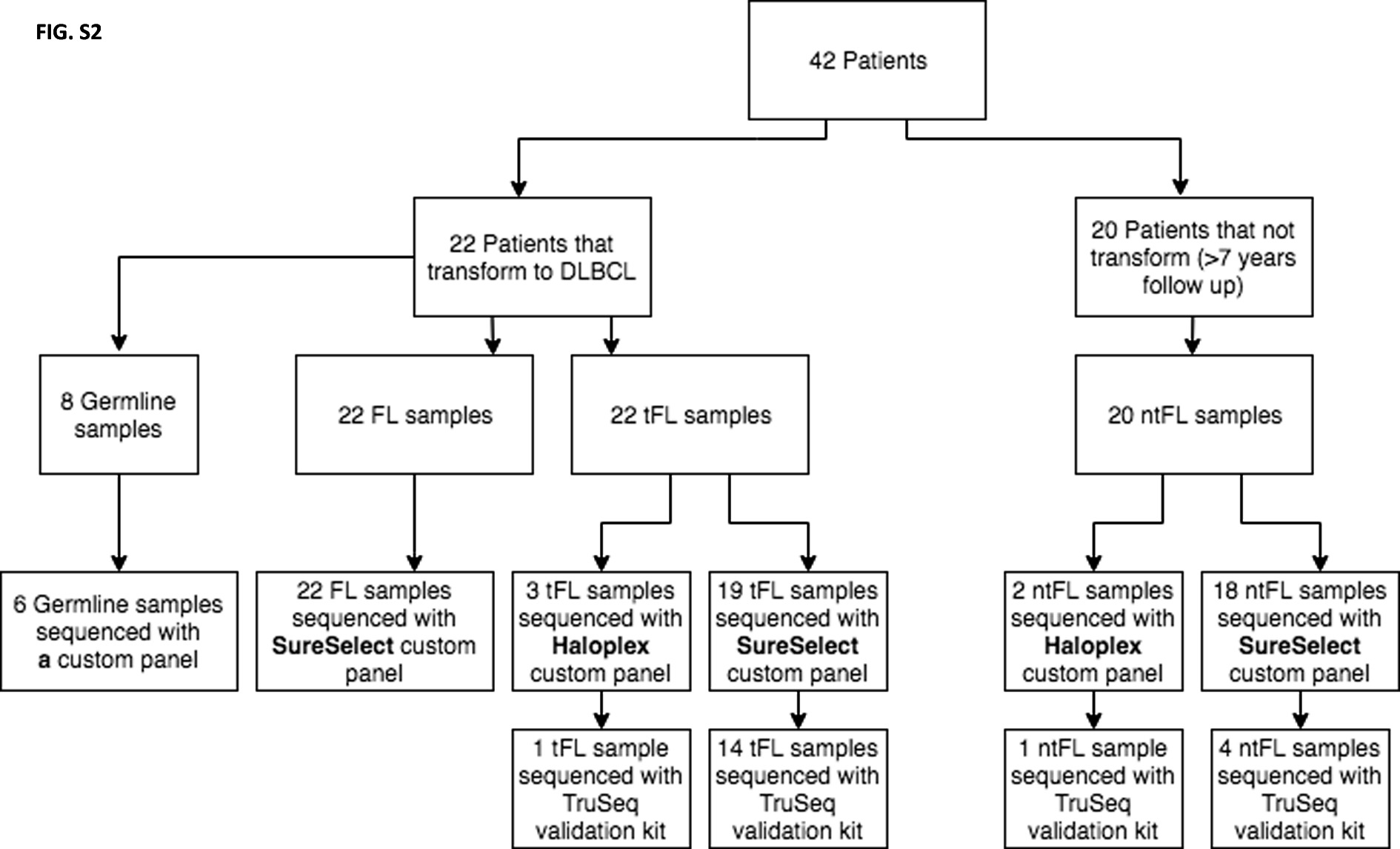

Supplement: S2 Fig — (TIF) [file pone.0212813.s002.tif]

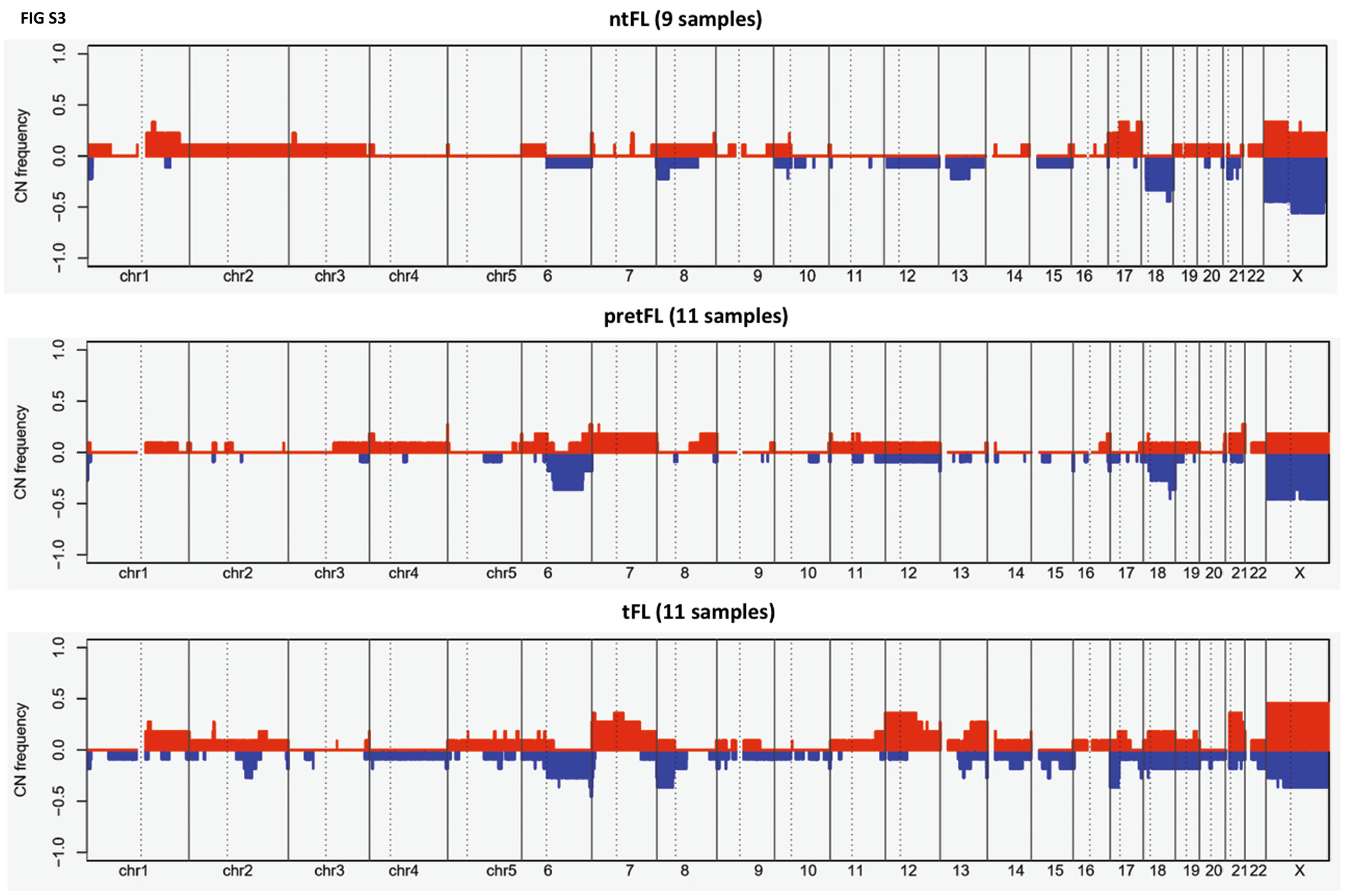

Supplement: S3 Fig — ntFL: non-transformed follicular lymphoma; pre-tFL: follicular lymphoma samples from transformed patients; tFL: diffuse large B-cell lymphoma samples from transformed patients. (TIF) [file pone.0212813.s003.tif]

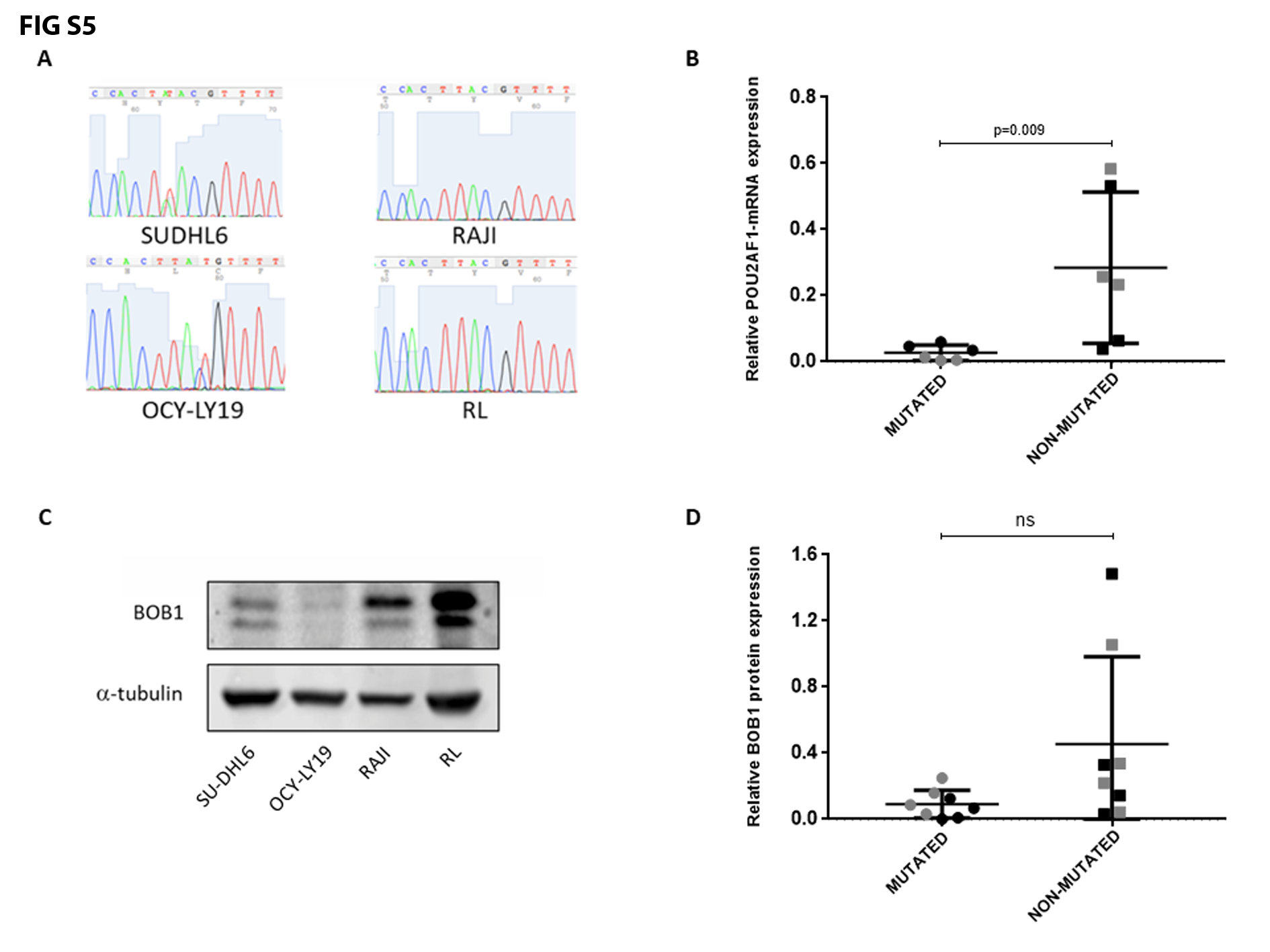

Supplement: S5 Fig — A) DNA sequences corresponding to the POU2AF1 gene in mutated cell lines (SU-DHL6, mutated in chr11: 111249884 T/A; and OCI-LY19 mutated in chr11: 111249886 C/T) and wild-type cell lines (Raji and RL). B) Quantitative RT-PCR analysis of POU2AF1-mRNA expression in the cell lines. Graphs represent the means of POU2AF1-mRNA levels and SDs, normalized with respect to SDHA, of three independent mRNA extractions; Mann Whitney two-tailed test, p = 0.009. C) Western blot analysis of BOB1 expression in the cell lines. D) Graphs show the means and SDs of quantified BOB1 protein levels normalized with respect to α-tubulin expression of four independent protein extractions; Mann Whitney two-tailed test, p = 0.065. ns: not significant; **: p<0.01. Grey circle: expression values corresponding to SU-DHL6 cell line; Black circle: expression values corresponding to OCI-LY19 cell line; Grey square: expression values corresponding to Raji cell line; Black square: expression values corresponding to RL cell line. (TIF) [file pone.0212813.s005.tif]

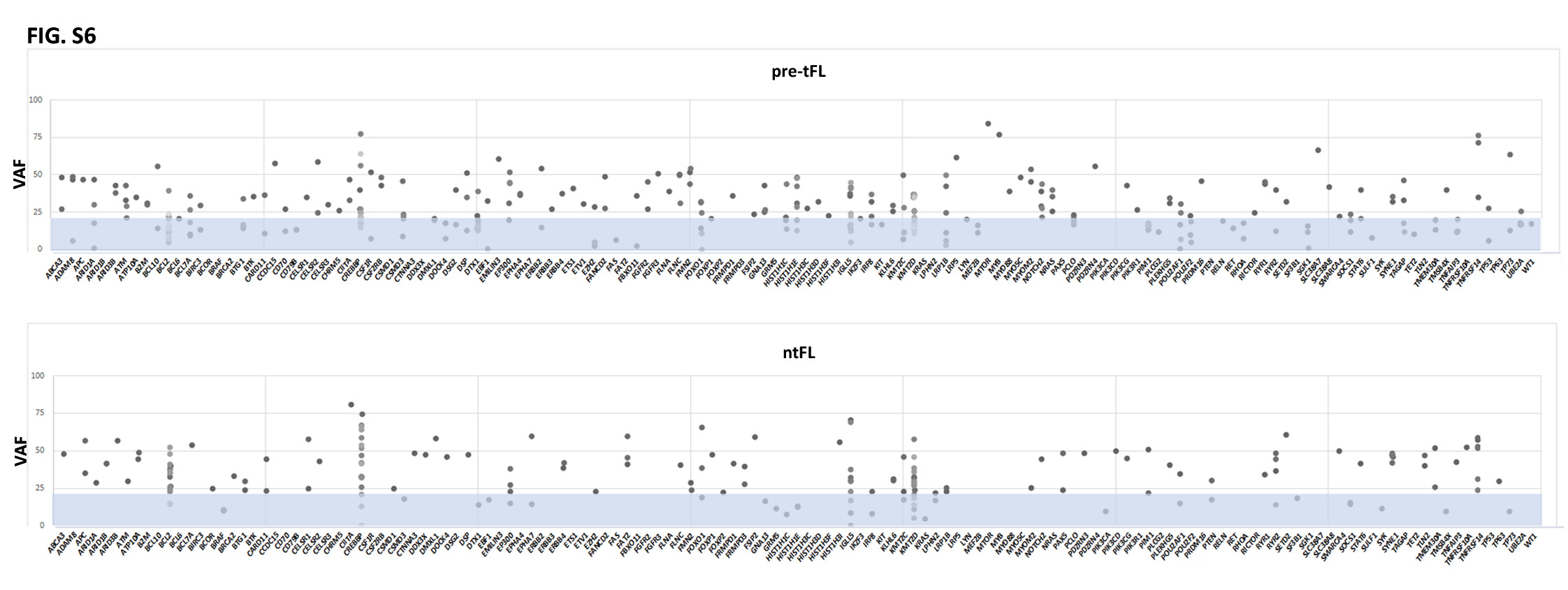

Supplement: S6 Fig — Blue boxes indicate VAFs < 20%. (TIF) [file pone.0212813.s006.tif]
