# Supplementary material for: Unraveling transformation of follicular lymphoma to diffuse large B-cell lymphoma
Source: PLoS One. 2019 Feb 25;14(2):e0212813. doi: 10.1371/journal.pone.0212813 (PMC6388933; doi:10.1371/journal.pone.0212813)

Figure S4 A. Clonal evolution of mutations from follicular lymphoma to diffuse large B-cell lymphoma. Divergent Evolution

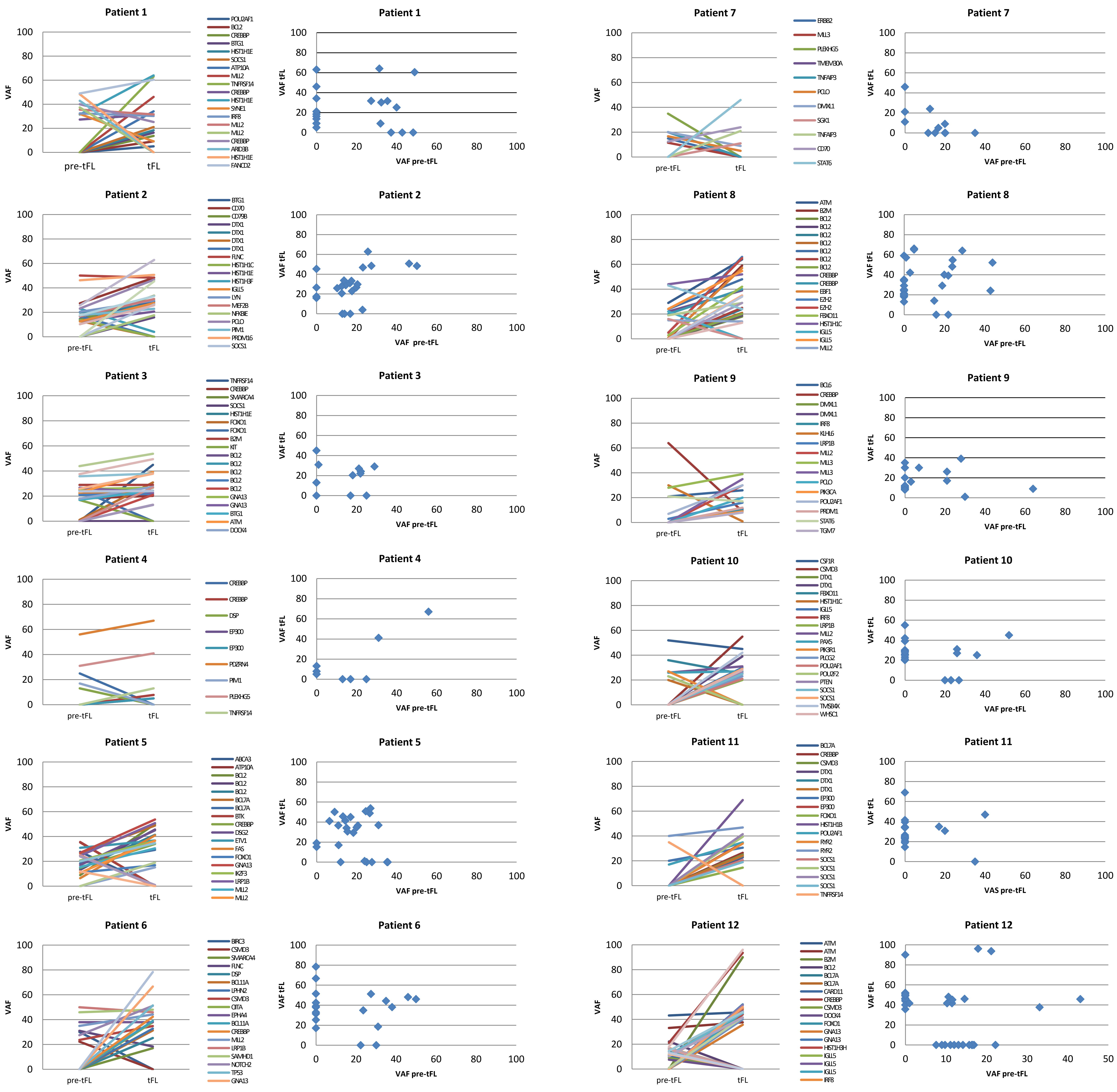

Figure S4 B. Clonal evolution of mutations from follicular lymphoma to diffuse large B-cell lymphoma. Linear Evolution

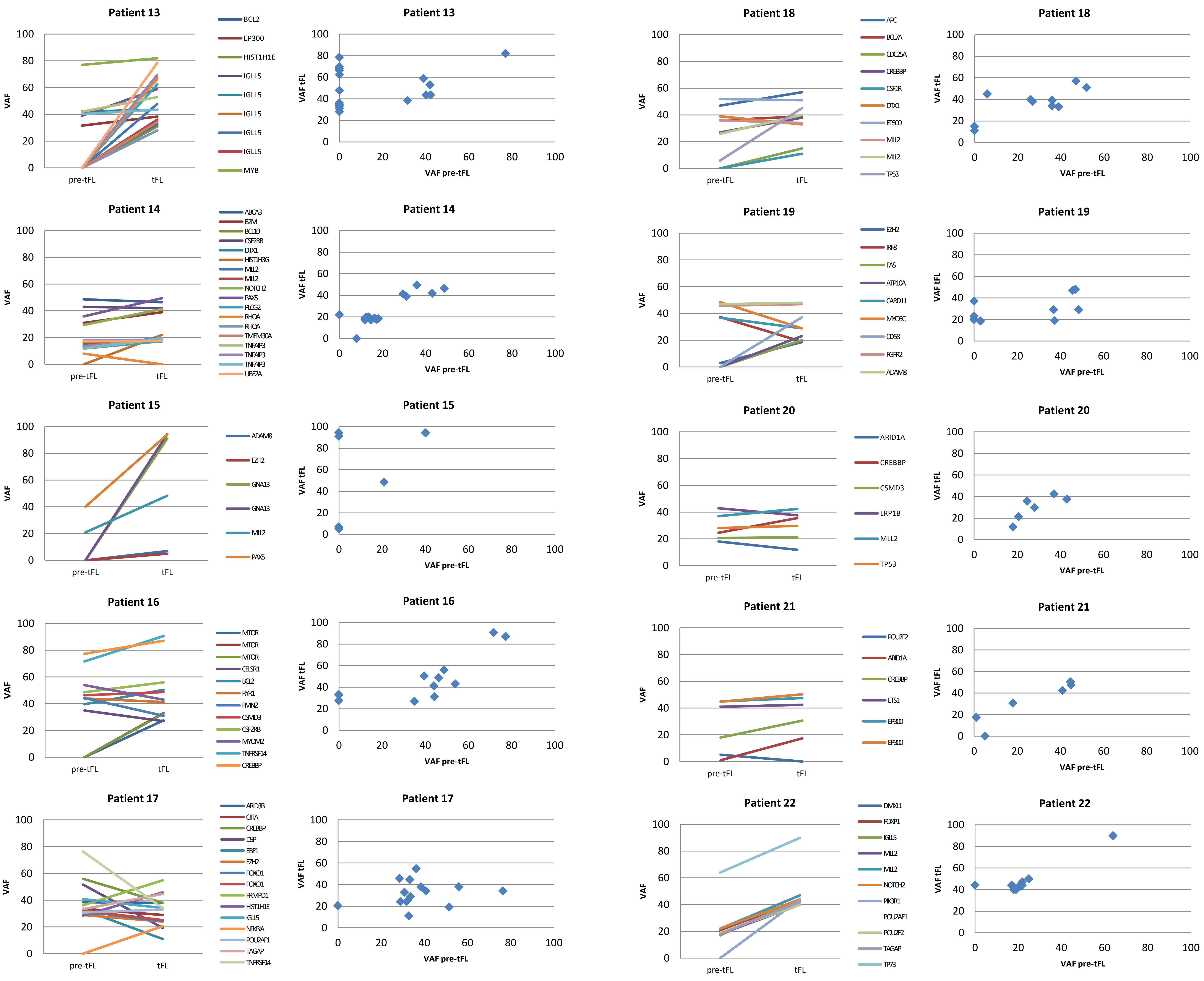

Supplement: S4 Fig — ntFL: non-transformed FL; pre-tFL: FL samples from transformed patients. (PDF) [file pone.0212813.s004.pdf]
